# Pathological evaluation of IVL-enabled modification and crossing in calcified chronic total occlusions

**DOI:** 10.1007/s12928-025-01216-4

**Published:** 2025-11-26

**Authors:** Yu Sato, Sho Torii, Gaku Nakazawa

**Affiliations:** 1 Division of Cardiology, Tokai University, 143 Shimokasuya, Isehara, Kanagawa 259-1193 Japan; 2 Division of Cardiology, Kindai University, Osaka, Japan

Intravascular lithotripsy (IVL) has emerged as a strategy for modifying heavily calcified plaques [1], which remain a major challenge in endovascular therapy [2]. The novel Javelin peripheral IVL catheter (Shockwave Medical, USA) was specifically designed for severely calcified stenotic lesions or chronic total occlusions (CTO) [3]. Approved in March 2025 in the United States, it enables precise calcium modification to facilitate lesion crossing without balloon expansion. We report the first application of the Javelin peripheral IVL catheter in human lower-limb arteries with severely calcified CTOs, confirmed by micro-computed tomography (microCT) and histological analysis.

Three calcified popliteal arteries were obtained from amputated limbs for an ex vivo study. Although a guidewire crossed all lesions, no device—including microcatheters, imaging catheters, or balloons—could pass. Treatment with the Javelin peripheral IVL catheter enabled successful device passage in all cases. The length of CTO lesions ranged from 21.6 to 36.1 mm, and the mean number of pulses needed to cross the lesions was 90 (range 45–150), while the device is designed to deliver up to 120 pulses.

MicroCT and histology confirmed the creation of a narrow channel penetrating dense nodular calcification with minimal surrounding disruption and no evidence of medial injury (Fig. 1). The absence of calcium fracture in surrounding sheet-like calcification may reflect the heterogeneous, mixed-content nature of the plaques, which can produce unpredictable fracture behavior under stress. These findings suggest that the Javelin peripheral IVL catheter facilitates lesion crossing by micro-disrupting calcified plaques, rather than dilating the lumen directly.

**Fig. 1:**
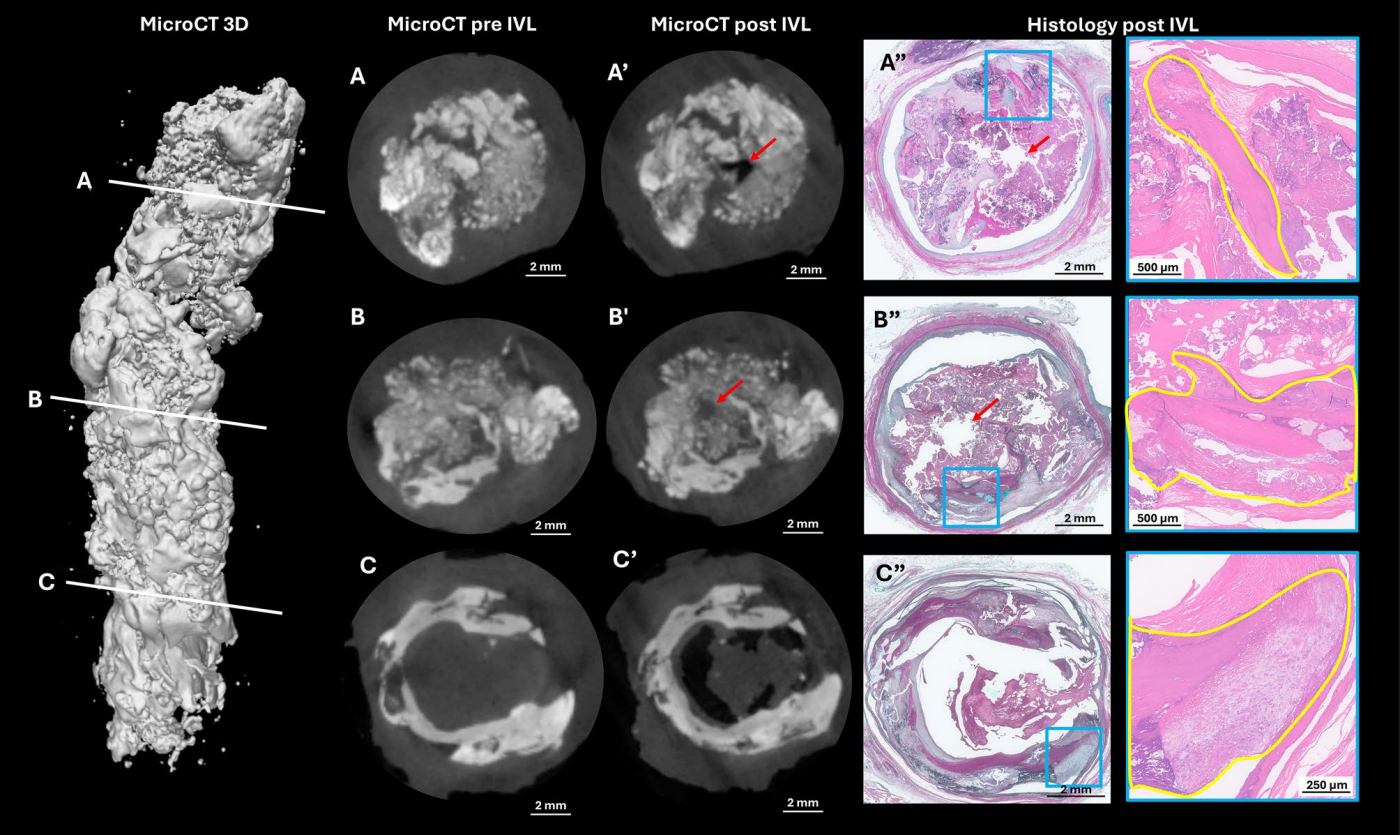
MicroCT (3D and cross-sections pre- and post-treatment) and matched histology of arteries treated with the Javelin catheter. White line **A**–**C** mark cross-sections **A**–**C** (pre-treatment) and **A**’–**C**’ (post-treatment). In images **A**’–**C**’, narrow channels 1 mm in size appears after treatment (red arrow in **A**’–**C**’ and **A**”–**C**”). Calcific nodules, sheet-like calcification (yellow highlighted), and surrounding tissues remain largely unchanged (blue boxed areas)

Traditionally, when no device crosses a lesion other than a guidewire, atherectomy has been the only option—but with risks such as vessel perforation and slow/no reflow. In contrast, the Javelin catheter may offer a safer alternative by modifying calcium while preserving adjacent vascular structures. The concept of “Javelin-enabled lesion crossing” has been emphasized in recent feasibility reports [3].

## Data Availability

Correspondence and requests for materials should be addressed to S.T.
